# Dexmedetomidine enables copper homeostasis in cerebral ischemia/reperfusion via ferredoxin 1

**DOI:** 10.1080/07853890.2023.2209735

**Published:** 2023-05-10

**Authors:** Qingduo Guo, Meina Ma, Hong Yu, Yuepeng Han, Dong Zhang

**Affiliations:** a Department of Anesthesiology, Cangzhou Central Hospital, Cangzhou, P.R. China; b Department of Anesthesiology, Hebei General Hospital, Shijiazhuang, P.R. China

**Keywords:** Cuproptosis, dexmedetomidine, cerebral ischemia-reperfusion

## Abstract

Excessive oxygen free radicals and toxic substances are generated in cerebral ischemia-reperfusion (I/R) process. Dexmedetomidine (DEX), a common anesthetic and sedative drug, can considerably boost glutathione (GSH), which has anti-copper influx effects. Focusing on cuproptosis, the mechanism of DEX in the I/R was revealed. Using the I/R rat model, the effects of DEX and the copper chelator D-penicillamine on cerebral infarct volume, copper levels, mitochondrial respiration and membrane potential, GSH content, and enrichment of cuproptosis functional proteins were examined. The involvement of ferredoxin 1 (FDX1) in the DEX regulatory pathway was verified by overexpressing FDX1 *in vitro*. DEX could significantly reduce cerebral infarction in rats, reduce copper levels, maintain mitochondrial functions, increase GSH, and reduce the content of key proteins related to cuproptosis. These aspects were replicated *in vitro* and revealed that FDX1 overexpression partially reversed the impacts of DEX. Together, cuproptosis occurs in the brain I/R process and DEX can enhance cell survival by blocking the primary pathway mediated by FDX1.

**KEY MESSAGES**
- Dexmedetomidine reduces cerebral infarction in the I/R rat models.
- Dexmedetomidine reduces cuproptosis in the I/R rat models.
- FDX1, an upstream of protein fatty acylation, mediates regulation of Dexmedetomidine.

## Introduction

Copper is an essential trace element, which plays a vital role in hemoglobin synthesis, bone formation, and the development of various internal organs [1,2]. It is also crucial to the functions of the central nervous system and the immune system [3,4]. Previous research has indicated that daily copper consumption is directly associated with the risk of hypertension, ischemic stroke, and other disorders. For each standard deviation increase in copper consumption, the risk of ischemic stroke rose by 20–30%. Surprisingly, this impact did not hold true for hemorrhagic stroke [5,6]. Around 800,000 people suffer a new stroke every year, the majority of which are ischemic strokes [7]. The current therapeutic guidelines for ischemic stroke propose restoring vascular blood and oxygen using medicines or surgery in order to limit brain tissue damage [8,9]. The restoration of cerebral blood flow, however, will be followed by a worsening of the pathological ­damage to brain tissue, known as cerebral ischemia-reperfusion injury (I/R). Excessive oxygen free radicals and toxic substances are generated as a pathogenic process [10].

High levels of copper ions can cause damage to vascular endothelial cells and neurons [11,12], and the internal mechanism is generally thought to be that copper ions trigger the accumulation of reactive oxygen species in cells and induce oxidative stress. The important protein copper metabolism domain containing 1 (COMMD1), which is involved in the metabolism of cellular copper homeostasis [13], was also significantly elevated in the brain tissue of aged MCAO rats, and the increase was more noticeable than in young MCAO rats. The increase of COMMD1 leads to the accumulation of copper ions in the cells, causing cell damage and even death [14]. The phenomenon and mechanism of cell damage and death caused by copper ions have been explored for a long time. The destabilization of cellular copper metabolism is a major cause, which manifests as the aggregation of fatty acylated proteins mediated by intracellular ferredoxin 1 (FDX1), loss of iron-sulfur cluster proteins, and aberrant mitochondrial respiration [15]. This type, which leads to proteotoxic stress and ultimately induces cell death, has been highlighted.

In GSE202659, SLC31A1 was dramatically elevated in the brain tissue of MCAO mice, as were other key markers of cuproptosis (lipoylated and fatty acylated proteins). The ubiquitous antioxidant stress factor glutathione (GSH) has been proven to protect against ischemic stroke [16], and GSH also a vital role in the protection of cells in the cuproptosis pathway [17]. As a consequence, molecules or medications that might activate GSH or raise its content may become promising research targets for preventing cuproptosis. Dexmedetomidine is a commonly used anesthetic and sedative drug in clinical practice, and it can considerably boost GSH levels [18]. Furthermore, molecular docking was carried out using the molecular structure of dexmedetomidine and the protein structure of SLC31A1, suggesting that the two may be capable of binding. Therefore, in addition to GSH, dexmedetomidine may influence the process of cuproptosis *via* direct impacts on SLC31A1.

This research will clarify the variations in the degree of cuproptosis in the rat I/R model and the PC12 cell OGD/R model, focusing on the expression levels of key regulators of copper ion homeostasis and cuproptosis. Combined with the anti-oxidation of dexmedetomidine, and further, through the anti-copper ion influx of GSH, the related mechanism was preliminarily clarified. This provides an experimental basis for further in-depth research on the cuproptosis mechanism of I/R and the following diagnosis and treatment.

## Methods and materials

### Animal models and handling

Sprague-Dawley rats (male, 8 weeks old, Charles River, Beijing, China) were housed in the vivarium with a natural light/dark cycle and ad libitum access to food and water. As previously described [19], the middle cerebral artery occlusion (MCAO) was used to induce focal cerebral ischemia. Using servo-controlled heating blankets, the body core temperature was maintained at ∼37 °C throughout the procedure. Rats were anesthetized with 2% isoflurane. After a midline neck incision, a nylon monofilament was inserted into the right common carotid artery and advanced through the internal carotid artery to block the origin of the MCA. One hour later, the occlusion wire was removed for reperfusion, and dexmedetomidine (DEX, 9 μg/kg), D-penicillamine (D-PCA, copper ion chelator, 11 mg/kg), and combined intravenous infusion were administered for 30 min simultaneously (*n* = 6) [20,21]. Rats were euthanized after 24 h.

### 2, 3, 5-triphenyltetrazolium chloride (TTC) staining

Rat brains were cut into 2 mm thick coronal sections. Sections were stained with 2% TTC for 20 min at 37 °C to visualize the infarct. The infarct volume was calculated by planimetry with ImageJ software.

### Cell culture

PC12 cells were used in *in vitro* research models, which were cultured in an incubator (37 °C, 95% air, and 5% CO_2_). Dulbecco’s modified eagle medium (DMEM) with 10% inactivated calf serum was applied for culture. To explore the mechanism, cells were transfected with plasmids (YouBio, Changsha, China) to overexpress FDX1. Briefly, PC12 cells were plated one day in advance. When the cell confluence reached ∼70%, the plasmids carrying FDX1 or empty plasmids (negative control) and transfection reagent were mixed evenly and added to the wells. Confirmation of transfection efficiency was performed 48 h after transfection. To mimic I/R, PC12 cells were undergone OGD/R treatment. Cells were cultured in serum-free DMEM in an incubator (37 °C, 95% N_2,_ and 5% CO_2_) for 4 h, treated with DEX, and re-oxygenated for a further 24 h.

### Copper level detection

The colorimetric quantification kit was provided by Scientifics Inc. (USA). The cleared brain tissue was quick-frozen in liquid nitrogen, taken out, ground into powder, and homogenized in the lysate. After centrifugation at 16,000 × g for 5 min, the supernatant was collected, the protein was quantified, and the anti-interference solution was replenished. The supernatant after re-centrifugation was co-incubated with the reducing solution for 5 min, and the absorbance (580 nm) was measured using a microplate reader (MD, Shanghai).

### Mitochondrial respiratory control rate (RCR)

Isolation of mitochondria was performed using a tissue mitochondrial isolation kit (Beyotime, Shanghai, China). Fresh brain tissue was homogenized in mitochondrial isolation reagent and centrifuged at 600 × g, 4 °C for 5 min. The obtained supernatant was centrifuged at 11,000 × g, 4° C for 10 min, and the precipitate was the mitochondria, which was then resuspended in storage buffer. Clark electrode instrument was applied to measure dissolved oxygen solubility in a 25 °C water bath. Fresh mitochondria were placed in the chamber until the rate of oxygen consumption stabilized. Supplementation with succinate to test low oxygen consumption rate (R IV) followed by ADP to stimulate high oxygen consumption rate (R III). RCR (R III/R IV) may indicate the mitochondrial capacity for oxidative phosphorylation.

### Mitochondrial membrane potential

Mitochondrial membrane potential was analyzed using a JC-1 detection kit (Yeasen, Shanghai). Frozen sections of fresh tissue or cell suspension were incubated with JC-1 staining working solution in the dark for 20 min. Upon centrifugation at 600 × g for 3 min to pellet the cells, the cells or tissue sections were washed twice with staining buffer and photographed under a fluorescent microscope (Leica).

### GSH content

The GSH content determination kit was provided by Biosharp Life sciences (Hefei, China). The extract liquid in the kit was supplemented to the tissue powder for homogenization, and the supernatant was gathered after centrifugation at 12,000 × g for 15 min. The supernatant was mixed with 5, 5′-dithiobis-2-nitrobenoic acid, and the absorbance (412 nm) was measured.

### Western blotting

Proteins were harvested from the tissue homogenate and PC12 cell lysate, quantified by Nano 300 and denatured by boiling. For lipoylated proteins, 10 μM reducing agent TCEP was added before boiling. After the separation and stacking gels were set up, electrophoresis was performed to separate the proteins. Through transfer sandwiches, hybridized membranes (Roche) were obtained. The membranes were blocked in skimmed milk and hybridized with appropriate primary antibodies and HRP-conjugated antibody (Proteintech, Wuhan, China). Blots were visualized with the ECL reagent (Millipore) and gray values were analyzed with ImageJ software. SLC31A1 antibody was provided by Invitrogen; ATP7B, LIAS, FDX1, SDHB, DLAT, GAPDH antibodies were provided by Proteintech; DLST and lipoic acid antibodies were provided by Abcam.

### Cell viability

Transfected or non-transfected cells underwent OGD/R and DEX treatment as abovementioned, and incubated with WST-8 reagent (Glpbio) for 2 h. The absorbance (450 nm) was measured.

### DLAT oligomerization

Indirect immunofluorescence was performed in fixed and permeabilized PC12 cells by incubation with DLAT antibody (CST, 1:100) overnight at 4 °C. This was followed by incubation with Alexa Fluor 488 secondary antibody for 1 h and counterstaining with DAPI for 6 min. Images were acquired under a fluorescent microscope.

### Statistical analysis

Data were presented and analyzed in the form of mean ± standard deviation in Prism 8.0. The Shapiro-Wilk test confirmed that the data were normally distributed, and differences were analyzed by one-way ANOVA and Tukey’s test. *p* < 0.05 means significance.

## Results

### DEX reduces cerebral infarction and copper ion levels in I/R rats

**Figure 1.**
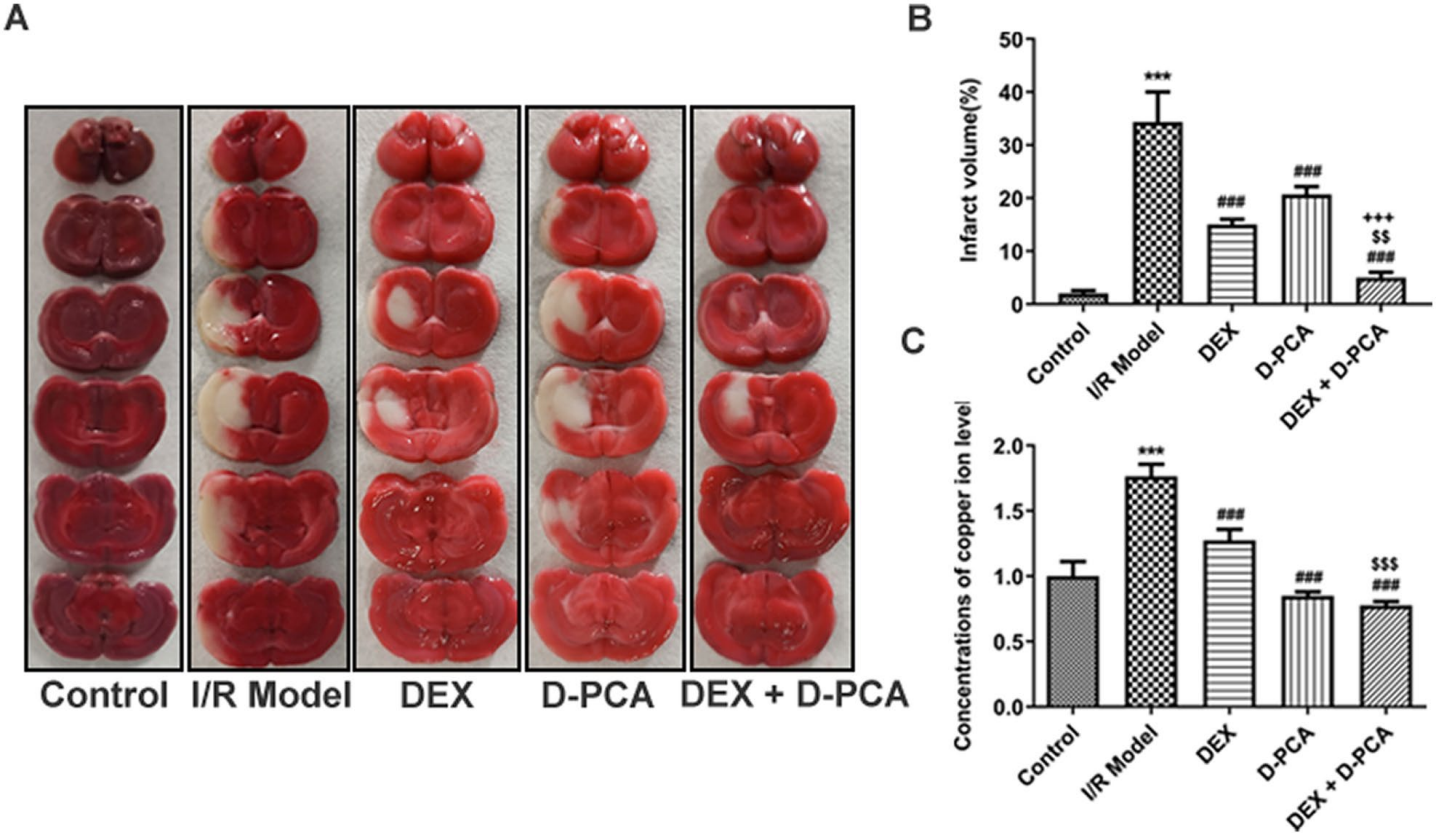
Dex reduces cerebral infarction and copper ion levels in I/R rat model (A) The brain sections were stained with TTC. (B) The cerebral infarct volume was quantified. (C) Relative concentration of copper levels in brain tissue. ***p < 0.001 vs. control; ^###^*p* < 0.001 vs. I/R model; ^$$^*p* < 0.01, ^$$$^*p* < 0.001 vs. DEX; ^+++^*p* < 0.001 vs. D-PCA.

Compared with the I/R group, Dex and D-PCA alone significantly reduced the cerebral infarct volume in rats, and such alterations were more notable in the combined drug group (Figure 1(A,B)). Quantified by colorimetry, both Dex and D-PCA administration significantly reduced copper levels in rat brain tissue compared to the I/R group. Meanwhile, the combined administration of Dex and D-PCA, compared with the Dex group, promoted a more distinct reduction in copper levels (Figure 1(C)).

### DEX regulates mitochondrial function and cuproptosis-related key factors in I/R rats

**Figure 2.**
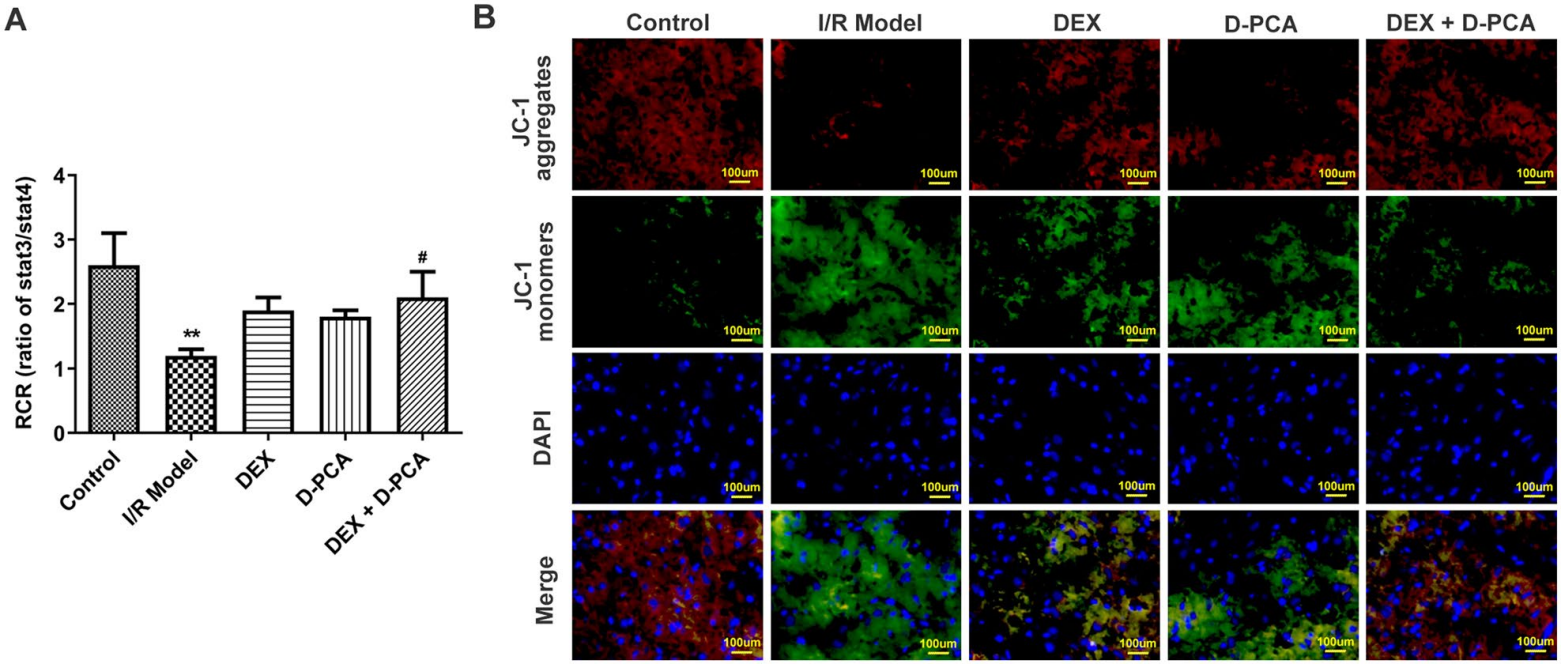
Dex maintains mitochondrial function (A) Fresh mitochondria were isolated and their respiratory control rates were examined. (B) Mitochondrial membrane potential was assessed by JC-1 probe. **p < 0.01 vs. control; ^#^*p* < 0.05 vs. I/R model.

RCR values were measured because destabilization of cellular copper metabolism can cause significant differences in mitochondrial respiration. RCR decreased significantly in the I/R group, implying damage to mitochondrial ATP synthesis and respiratory disorder. Both DEX and D-PCA administration can alleviate mitochondrial damage, and the effect of the combined treatment was more effective (Figure 2(A)). Mitochondrial membrane potential can also reflect mitochondrial health and cell status, and the decrease of membrane potential is a landmark event in the early stage of cell death. JC-1 aggregates decreased and monomers increased in the I/R group, indicating a decrease in membrane potential. The administration of DEX and D-PCA could reduce the decline of membrane potential to a certain extent, and the membrane potential was close to the state of the control group when combined administration (Figure 2(B)).

**Figure 3.**
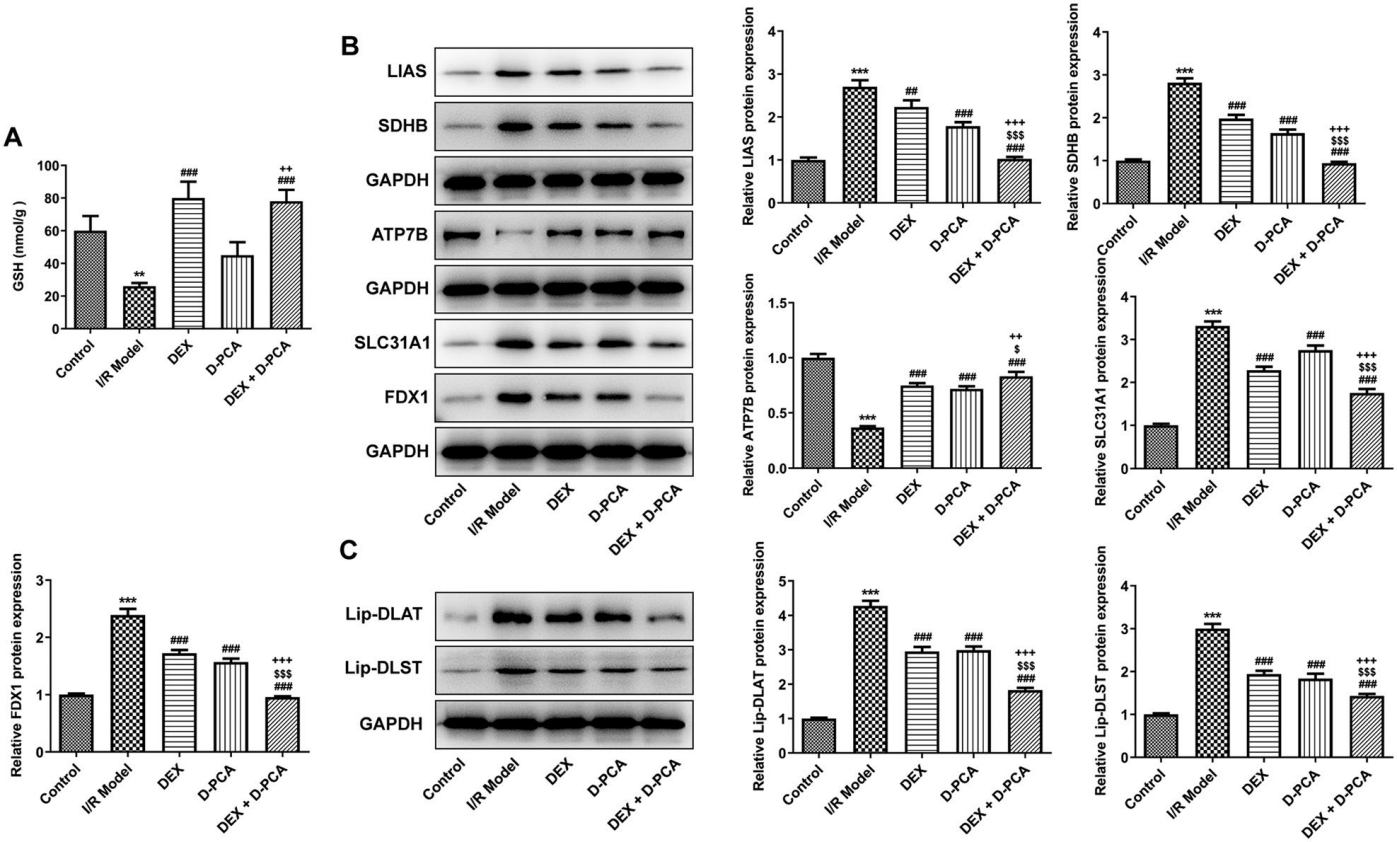
Dex regulates the key factors related to cuproptosis (A) The GSH content in the brain tissue was detected by a kit. (B) Contents of cuproptosis-related proteins were determined using western blotting. (C) Contents of lipoylated proteins were determined using western blotting. **p < 0.01, ***p < 0.001 vs. control; ^##^*p* < 0.01, ^###^*p* < 0.001 vs. I/R model; ^$^*p* < 0.05, ^$$$^*p* < 0.001 vs. DEX; ^++^*p* < 0.01, ^+++^*p* < 0.001 vs. D-PCA.

GSH is a crucial anti-oxidative stress factor that protects cells in the cuproptosis pathway, and its content was down-regulated in the I/R group. In contrast, DEX significantly increased the level of GSH, whereas D-PCA administration alone did not cause a marked difference in GSH. Compared with the D-PCA group, combined treatment can significantly increase the GSH content (Figure 3(A)). Afterward, cuproptosis-related functional proteins were determined using western blotting. SLC31A1, FDX1, LIAS, and SDHB showed increased levels in response to I/R, accompanied by decreased ATP7B levels. The administration of DEX and D-PCA can partially alleviate the fluctuation of these protein contents, and the combined administration can obtain a better recovery (Figure 3(B)). Lip-DLAT and lip-DLST were both increased in the I/R group, DEX and D-PCA had similar effects on their content, and the combination of the two caused a more obvious decrease (Figure 3(C)).

### Mediation of FDX1 in DEX regulation *in vitro*

**Figure 4.**
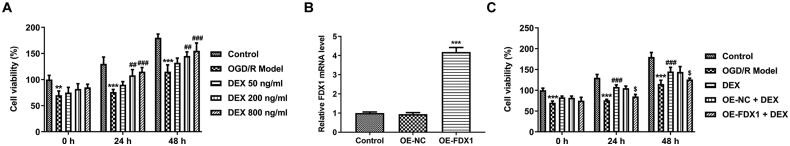
Effects of Dex on PC12 cell viability (A) Cell viability was measured with the CCK8 assay. (B) Transfection efficiency was checked by RT-qPCR. (C) The effect of FDX1 overexpression on cell viability was assessed. **p < 0.01, ***p < 0.001 vs. control; ^##^*p* < 0.01, ^###^*p* < 0.001 vs. OGD/R model; ^$^*p* < 0.05 vs. OE-NC + DEX.

**Figure 5.**
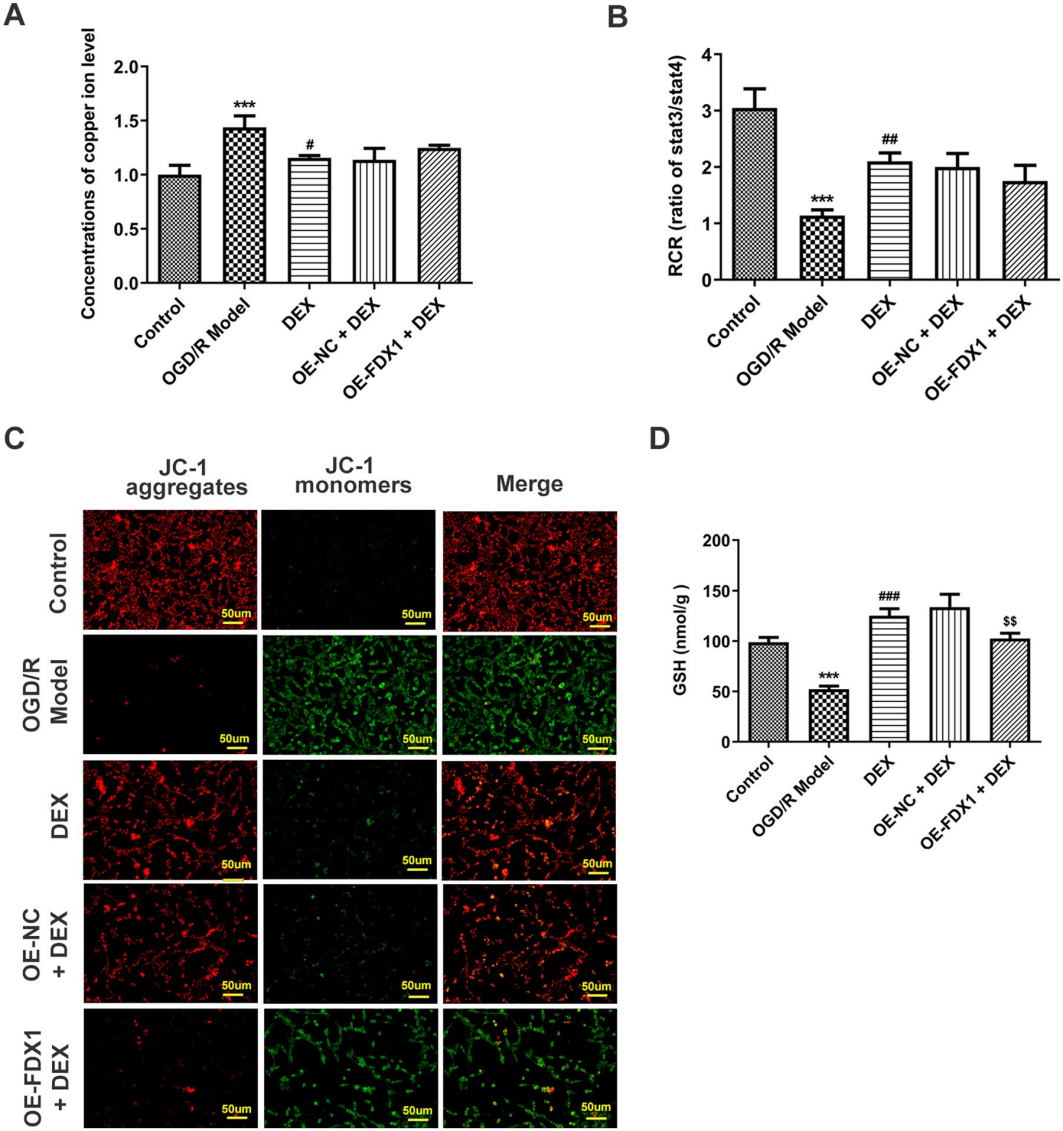
Mediation of FDX1 in mitochondrial function (A) Relative concentration of copper levels in PC12 cells. (B) Mitochondrial respiratory control rates were examined. (C) PC12 cell mitochondrial membrane potential was assessed by JC-1 probe. (D) The GSH content in the PC12 cells was detected by a kit. ***p < 0.001 vs. control; ^#^*p* < 0.05, ^##^*p* < 0.01, ^###^*p* < 0.001 vs. OGD/R model; ^$$^*p* < 0.01 vs. OE-NC + DEX.

**Figure 6.**
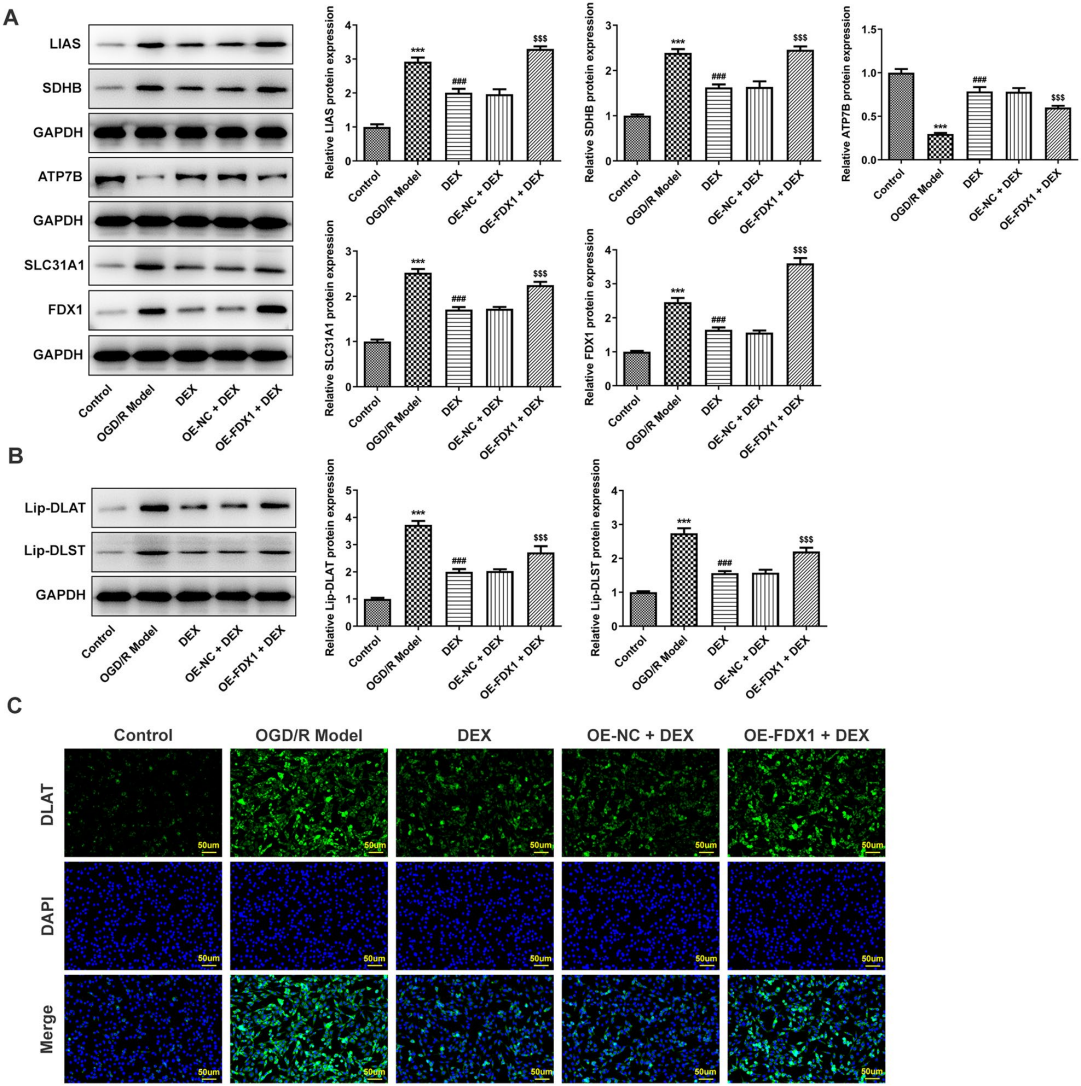
Mediation of FDX1 in key factors related to cuproptosis (A) Contents of cuproptosis-related proteins in PC12 cells were determined using western blotting. (B) Contents of lipoylated proteins were determined using western blotting. (C) DLAT oligomerization was assessed with immunofluorescence. ***p < 0.001 vs. control; ^###^*p* < 0.001 vs. OGD/R model; ^$$$^*p* < 0.001 vs. OE-NC + DEX.

In the *in vitro* research model, OGD/R induced a decrease in cell viability, and DEX could increase cell viability in a concentration-dependent manner (Figure 4(A)). Since FDX1 mediates key steps in the cuproptosis process, including copper ion reduction and aggregation of lipoylated proteins, PC12 cells were transfected to overexpress FDX1 (Figure 4(B)). After cells overexpressing FDX1 were treated with a moderate dose of DEX, their viability was reduced compared with the OE-NC + DEX group (Figure 4(C)). In addition, DEX treatment significantly reduced the level of copper ions in cells, whereas FDX1 overexpression had no significant effect on the content of copper ions (Figure 5(A)). In terms of RCR, DEX treatment made it significantly reduced, and FDX1 overexpression slightly reduced it, but again not significantly (Figure 5(B)). However, FDX1 overexpression could markedly reduce mitochondrial membrane potential (Figure 5(C)) and GSH content (Figure 5(D)). In the results of cuproptosis-related functional proteins, compared with the OGD/R model group, DEX decreased the contents of SLC31A1, FDX1, LIAS, and SDHB, and increased ATP7B, while FDX1 overexpression could partially reverse the effect of DEX (Figure 6(A)). Moreover, FDX1 overexpression promoted the increase of lip-DLAT and lip-DLST content, which destroyed the inhibitory effect of DEX (Figure 6(B)). The binding of copper to lipoylated proteins leads to protein oligomerization. Immunofluorescence results demonstrated that OGD/R treatment induced DLAT oligomerization, and DEX significantly inhibited this phenomenon, and FDX1 overexpression hindered the effect of DEX on DLAT oligomerization (Figure 6(C)).

## Discussion

Copper is a cofactor for several critical metabolic enzymes that drive a wide range of physiological processes. Copper levels must be maintained within a narrow range to guarantee proper metabolic processes [22,23]. To maintain copper homeostasis at the cellular level, the intracellular copper content is regulated by a complex network of copper-dependent proteins, including copper enzymes, copper chaperones, and membrane transporters [24]. These proteins collaborate to coordinate the import, export, and intracellular utilization of copper to maintain intracellular copper levels within specific ranges, therefore avoiding the consequences of copper overload and copper insufficiency [25,26]. SLC31A1, a high-affinity copper transporter, is responsible for the majority of copper uptake into cells, and accumulating evidence suggests that SLC31A1 is essential for copper transport to certain organs/tissues [27,28]. Mice with SLC31A1 depletion, for example, had reduced copper in cardiac tissues, indicating that SLC31A1 is a primary factor driving cardiac copper homeostasis [29]. SLC31A1 inhibition can thereby modulate the cuproptosis pathway by influencing copper absorption. From a macro viewpoint, whereas copper chelators dramatically reduced copper ion absorption in cells, DEX was superior in lowering SLC31A1 and boosting GSH content, which may be owing to the cascade of signals induced by DEX.

Given that the reductase FDX1 facilitates the conversion of Cu^2+^ to more toxic Cu^+^ and protein lipoylation [15], we focused on the downstream FDX1 of SLC31A1 in subsequent *in vitro* studies. FDX1 was lower in DEX-treated rat brain tissue and PC12 cells than in the model group. The reversal experiment (FDX1 overexpression) indicated that FDX1 was an important regulator of DEX on cuproptosis. Specifically, FDX1 overexpression had a substantial effect on mitochondrial membrane potential, GSH content, lipoylated protein, and lipoylated protein content. During reperfusion, reductase converts oxidized GSH to GSH, which facilitates the clearance of free hazardous chemicals [30,31], and the inhibitory impact of FDX1 on GSH causes intracellular oxidative stress. Multiple studies have shown that oxygen free radicals play an important role in cerebral infarction and cell death in cerebral I/R [32,33], and reoxygenation after vascular occlusion offers substrates for numerous enzyme oxidation processes and mitochondrial oxidative phosphorylation, culminating in ATP [34]. Although there was no evident correlation between FDX1 and mitochondrial oxidative phosphorylation, the damage induced by OGD/R and the improvement effect of DEX did exist. In addition, FDX1 overexpression has no significant influence on the copper content of PC12 cells. We speculate that because FDX1 is enriched in the cell and downstream of SLC31A1, it cannot produce large enough negative feedback on the SLC31A1 function. Alternatively, in pathological circumstances, GSH depletion enhances copper influx, which counteracts the inhibitory impact of DEX. As can be noted, there are several limitations in this study, and the above conjectures require further exploration.

## Conclusion

In summary, based on the rat I/R model and the PC12 cell OGD/R model, this study discovered that the cuproptosis pathway occurs in the brain I/R injury process. DEX can enhance cell survival by blocking the primary cuproptosis pathway mediated by FDX1. These findings suggest the antioxidant effect of DEX and offer a foundation for further research into the mechanism of cuproptosis in stroke or I/R injury.

## Data Availability

The datasets generated during and/or analysed during the current study are available from the corresponding author on reasonable request.
